# The two envelopes method for active learning

**DOI:** 10.3205/zma001551

**Published:** 2022-07-15

**Authors:** Moshe Y. Flugelman, Robert M. Glueck, Doron Aronson, Avinoam Shiran

**Affiliations:** 1 Lady Davis Carmel Medical Center, Department of Cardiovascular Medicine, Haifa, Israel; 2 Technion Israel Institute of Technology, Rappaport Faculty of Medicine, Haifa, Israel; 3 Rambam Medical Center, Department of Cardiology, Haifa, Israel

**Keywords:** active learning, small group learning, peer learning, clinical reasoning

## Abstract

**Purpose:**

Active learning improves knowledge acquisition and provides medical students with learning habits that become an integral part of their behavior. As an integral element of our institution’s transition from a lecture hall teaching culture to active learning, the current project, conducted with fourth year students, aimed to examine the effects of the two envelopes method of teaching on students’ knowledge.

**Method:**

The class of 120 students was divided into 12 groups of 10 students each. Six experienced senior cardiologists were assigned to teach the 12 groups. When the students arrived at the classroom, they received two envelopes. Students were instructed to open the first envelope and answer a 10-question test in 15 minutes. After completing the test, they returned the tests to the envelope, sealed it, and then opened the second envelope which included the same test and relevant patient information. They then spent the next 30 minutes discussing the test as a group and familiarizing themselves with the patients’ case histories and clinical data. After completion of the group discussion, the tutor entered the room for a two-hour discussion of the patients’ disease entities including the anatomy, physiology, pathology, clinical presentation, diagnostic measures, and potential therapies.

**Results:**

We compared grades and standard deviations of grades between two classes: one learned in the lecture hall format (2018) and the other learned employing the two-envelopes method (2019). There was a non-statistically significant trend toward better grades with reduced dispersion of grades in the class that learned with the two-envelope method.

**Conclusions:**

We describe a novel method for active learning that enhances self-learning and peer learning, and we observed better knowledge acquisition and reduced knowledge dispersion that were not statistically significant.

## Zusammenfassung

**Zweck der Studie:**

Aktives Lernen verbessert die Aneignung von Wissen und vermittelt Medizinstudierenden Lerngewohnheiten, die zu einem integralen Bestandteil ihrer Verhaltensweise werden. Das aktuelle Projekt wurde mit Studierenden im vierten Studienjahr durchgeführt und diente als wichtiges Element bei der Umstellung unserer Einrichtung von der bisherigen vorlesungsbasierten Lehrkultur zu einer aktiven Lernkultur und dazu, die Auswirkungen der Zwei-Umschläge-Methode auf die Kenntnisse der Studierenden zu untersuchen.

**Methode:**

Der Kurs mit 120 Studierenden wurde in 12 Gruppen mit je 10 Studierenden aufgeteilt. Sechs erfahrene Kardiologen wurden den 12 Gruppen als Lehrkräfte zugeteilt. Im Lehrsaal erhielten die Studierenden zwei Umschläge. Ihnen wurde aufgetragen, den ersten Umschlag zu öffnen und einen Test mit 10 Fragen innerhalb von 15 Minuten zu beantworten. Nach Abschluss des Tests steckten sie die Testbögen wieder in den Umschlag, verschlossen diesen und öffneten dann den zweiten Umschlag, in dem sich der gleiche Test mit relevanten Patienteninformationen befand. Sie verbrachten die nächsten 30 Minuten damit, den Test in der Gruppe zu besprechen und sich mit den Fallbeschreibungen und den klinischen Daten der Patienten zu befassen. Nach Abschluss der Gruppendiskussion betrat eine Lehrkraft den Raum für eine zweistündige Besprechung der Krankheitsbilder der Patienten, einschließlich der Anatomie, Physiologie, Pathologie sowie der klinischen Erscheinungsbilder, diagnostischen Maßnahmen und potenziellen Therapien.

**Ergebnis:**

Wir verglichen die Ergebnisse und Standardabweichungen durch die Noten der beiden Kurse. Der eine Kurs wurde in Form von Vorlesungen unterrichtet (2018), der andere Kurs erlernte die Anwendung der Zwei-Umschläge-Methode (2019). Im Kurs mit der Zwei-Umschläge-Methode waren die Noten ohne statistische Signifikanz tendenziell besser und die Notenstreuung tendenziell kleiner.

**Fazit:**

Wir beschreiben eine neuartige Methode für aktives Lernen, die eigenständiges Lernen und kollegiales Lernen in der Gruppe (peer learning) fördert, und beobachteten einen besseren Erwerb von Wissen und geringere Abweichungen im Wissensstand, ohne statistische Signifikanz.

## Background

Teaching medicine has been an ever-changing challenge since the sentinel publication of Flexner [1], [2], [3]. Multiple methods have been employed over the years to improve knowledge and behaviors of future physicians. Improved understanding of learning processes and the realization that teaching methodology must adapt to new technology and the psychological and social changes of medicine, students and society, has promoted active learning as a leading theme in medical schools [4], [5], [6], [7], [8], [9], [10], [11], [12]. 

Transferring responsibility for learning to the students requires changes in the attitudes of both students and teachers. Methods such as flipped classrooms and just-in-time learning have gained popularity in many medical schools as part of the active learning theme [13], [14]. The introduction of active learning methodologies to teachers (kindergarden-12^th ^grade) by Chi et al was based on the so called ICAP model [15] which differentiated students’ engagement in learning based on their behaviors. Changing from traditional learning methods to active learning methodology requires changes in resource allocation and a partnership of all stakeholders including students, teachers and educational systems regulators [5], [15]. 

We initiated a faculty-wide effort to introduce active learning to our curriculum which is currently based on lecture-embedded knowledge transfer with a minor element of active learning, mostly in laboratories. In this manuscript we describe a novel method that melds active learning with peer learning of heart valve-related diseases as part of the cardiology course, and we tested its effect on student knowledge acquisition in the final examination. This novel method, namely the two envelopes method, transfers responsibility for learning to the students by assigning compulsory reading and autonomous peer discussion to small groups. In the present study, we hypothesized that students’ knowledge would improve with the two envelopes method as compared with traditional lecture-based pedagogy. 

## Methods

### The 2 envelopes method

Fourth year students in our 6-year medical school program study the main subjects of internal medicine integrated with system pathology and clinical pharmacology. As part of learning cardiology, the topics of valvular disease and rheumatic heart disease were chosen for the introduction of active learning. The other topics of cardiology are taught in the classroom, and coronary artery disease is taught in the hospital using a problem-based learning methodology. 

As part of the active learning protocol the students were asked to read about five disease entities in Harrison’s Principles of Internal Medicine including mitral stenosis, mitral regurgitation, aortic stenosis, aortic regurgitation, and rheumatic heart disease. The class of 120 students was divided into 12 groups of 10 students each. Six experienced senior cardiologists were assigned to teach the groups. The teachers met and reached a consensus regarding the central learning points for each disease entity prior to meeting the groups. Five detailed patient histories relating to each of the valvular pathologies and rheumatic heart disease were provided to the teachers as materials for the group learning sessions. 

The introduction of the novel learning method was discussed with the student union prior to its introduction, and the students received a letter describing their learning assignment and the way the group meeting would be conducted, including the two envelopes method. Three subjects were discussed in the first session and the remaining two disease entities in the second session. 

When the students arrived at the faculty, each group was reintroduced to the two envelopes method. They then received two envelopes which included a short test on the subject syndromes. The students were allowed 15 minutes to complete the test in the first envelope. The tests were then returned to the first envelope which was sealed. The students subsequently opened the second envelope which included the same test as well as patients case studies including history, physical examination, laboratory and imaging evaluation, treatment and outcome of the syndromes that were the subject of the meeting. The students then spent the next 30 minutes discussing the test as a group and familiarizing themselves with the patient details. After completion of this group conference, the tutor-instructor entered the room for a two-hour discussion of the disease entities including anatomy, physiology, pathology, clinical presentation, diagnostic measures, and therapies. 

The same methodology was used for the two other disease entities. After completion of the 3 week cardiology course, the students completed a test which included multiple choice questions (MCQ) and a comprehensive integrative puzzle (CIP) on all cardiology topics including the five entities studied using the two-envelope methodology. The CIP included five different clinical diagnoses, such as acute myocardial infarction and mitral stenosis. The students were requested to complete the following sections regarding each clinical diagnosis: patient’s complaints, physical findings, laboratory findings, pathological findings, and course and treatment. The students were asked to choose the best of five possible answers for each section. Once the CIP was completed, a comprehensive description of the diagnosis should become evident. 

We identified the questions in the test related to the five disease entities taught using the two envelopes method, studied the number of correct answers and compared these results to previous years’ tests in which the five disease entities were taught using a standard frontal lecture format. 

#### Statistical analysis

We analyzed the average grades of the students in 2018 when the teaching of valvular disease was presented in lecture halls. We documented the average grades on questions related to valve disease and the standard deviations both in the multiple-choice part and in the comprehensive integrative puzzle. We then documented the average student grades from 2019 when valvular disease was taught using the 2 envelopes methods. We compared the grades for questions related to valvular disease and questions related to non-valvular disease both in the MCQ and CIP portions of the examinations for the two years. 

Grades data were tested for normality using the Shapiro-Wilk test and equality of variance using the Levene’s test. Because the Shapiro-Wilk test indicated that grades were not normally distributed, grades are presented as medians (interquartile range [IQR]) and compared with the Mann-Whitney U test. Differences were considered statistically significant at the 2-sided P<0.05 level. Statistical analyses were performed using the Stata Version 16.1 (College Station, TX). 

## Results

In 2018 120 students were tested and in 2019 122 students were tested. A summary of the number of questions in valve-related diseases and the average grades in 2018 and 2019 are presented in table 1 (Tab. 1). In 2019 we increased the number of valve-related questions. 

When we compared the grades of the valve related and non-valve related questions in 2018 and 2109, we could not find significant statistical differences. There was a tendency for higher grades in valve-related MCQ questions and narrower variances in 2019 (see table 2 (Tab. 2)), as was the case for valve-related questions in the CIP part of the test (see table 2 (Tab. 2)) but these differences were not statistically significant. 

## Discussion

In the current manuscript we describe a novel method to introduce active learning to 4^th^ year medical students. This method promoted individual active learning and peer learning of 4 heart valve disease entities and rheumatic heart disease. Based on the final examinations, we observed a trend, which was not statistically significant, for better knowledge acquisition based on the two-envelope methodology, both in MCQ and CIP questions. We also observed lower dispersion of grades as an indicator of better knowledge dispersion using the two envelopes method. 

The changing methodology and culture of learning over the last several decades reflect the following paradigms:

1. In light of the current explosion of knowledge, students must be given the intellectual tools to seek and identify valuable information rather than merely acquire it in classes.
2. Technological changes have changed the interface between individuals and the traditional teaching hierarchy.
3. Social changes in society have created different expectations from individuals whether they are students, teachers or professionals and have promoted nonconventional behaviors, and
4. better understanding of learning mechanisms have given rise to novel teaching methodologies [4], [5], [6], [7], [8], [9], [10], [11], [12].

Currently, many topics are taught in lecture halls in our faculty and the students are tested on the topic at the end of the semester. As part of the effort to change teaching methodology from frontal lectures to active learning we developed the two-envelopes method. The two-envelope method that we describe encompasses several aspects of modern educational theory, and updates methodologies with transfer of learning responsibility to the student. Providing a specific compulsory reading task and a short test makes all students study the relevant material on their own before class. It was recently shown that tests improve learning beyond simply testing the information taught [16]. After completion of the test, the students receive the second envelope and are asked to reconsider the test questions as a group. This peer activity improves learning, and both promotes motivation to learn in weak students and improved self-confidence in strong ones. Autonomous (students only) discussion also transfers learning responsibility to the students by creating a peer-managed environment that encourages open discussion by all participating students. Knowledge sharing is also an aspect of education that is important to the students for future professional behaviors [17], [18].

After completion of the test, the students are asked to discuss the cases provided in the second envelope. In this part of the process, they are required to integrate learned data with patient histories. Again, group dynamics improves motivation and self confidence in the students [9]. After completion of group learning the addition of the tutor to the group is time efficient, as the students have already studied the subject at three different levels: self-learning, group learning, and integration of information to patient case studies. 

Learning in a small group and using a patient case study as the basis for discussion provides an invaluable platform to develop knowledge, as well as an opportunity to develop clinical reasoning and pattern recognition [19], [20]. Both clinical reasoning and pattern recognition are important aspects of becoming a competent physician. 

An indirect educational benefit of the program is that the students conducted the first part of the encounter, including the test, without faculty supervision. This provides a strong message that we trust our students and expect ethical behavior from them. 

A successful program such as the one we describe requires a dedicated and capable group of tutors. We selected experienced cardiologists and gathered all tutors for a detailed project description and discussion. We highlighted the importance of engaging all the students in the discussion and conducting the encounter as a creative conversation and not a lecture. We asked the tutors to elucidate the important points of each syndrome, and then asked all of them to teach and emphasis these points. Reaching consensus on what is important secures standardized teaching in all the groups. While Chi et al. [15] focused in their project on the teachers, our task was simpler, based on the relatively small scale of operation and the teachers we chose. Physicians have taught students at the bedside using active learning methodologies for centuries [21], [22], instinctively employing effective pedagogical methodologies that have gained wide popularity in recent years. The lack of statistical significance in our results can be explained by the fact that grades in cardiology were high over the last two decades as cardiology was the most popular course during the fourth year with high lecture attendance and student engagement. Improving an already excellent course is challenging, and our main goal was to introduce active learning methodology. Successful introduction of new methodologies in education is always difficult--especially in medicine, where students and teachers are often conservative. Changes in teaching methods, such as those required during the COVID-19 pandemic, as well as during less demanding circumstances, should be carefully evaluated [23]. Assessment of new teaching methods should include evaluation both by students and teachers, and rigorous assessment of knowledge acquisition using both qualitative and quantitative standardized metrics [24], [25], [26]. 

Another important aspect of the two envelopes method is that students realize they can learn complex subjects on their own, and that the knowledge they gain is a direct result of their effort and interaction with their peers. During student reviews at the end of the course, many students shared with us their sense of accomplishment, but this aspect was not studied systematically. Such success, as evidenced in the present study by a trend toward better final test grades, promotes self-efficacy and provides reassurance that our students are indeed capable of profiting from active learning [27]. 

Active learning theory which is based on better understanding of how we learn and how we develop cognitive skills, encompasses many methods. The two envelopes method engages the students actively in learning and teaches them to reflect on what they have learned by allowing them to discuss the knowledge gained with peers and with the tutor. It also engages problem-solving and critical thinking skills while learning the patient cases provided in the second envelope, initially on their own, then with their peers and finally with the tutor. 

As previously reported in other manuscripts describing transitions to active learning, the students expressed a high level of satisfaction, as did the tutors, but this was not tested systematically. 

Study limitations include comparison of test results of different classes that learned the five disease entities in the classroom using conventional lectures and the relatively small number of questions focused on the 5 disease entities. 

Additional studies testing the two envelopes method should be conducted. Students’ responses to learning using this method should be included [24], [25] in future studies and the method should be evaluated in other medical schools. 

## Conclusion

We introduce a novel method of active teaching that encompasses multiple innovative pedagogical methods. The two envelopes method as employed in the present study resulted in a nonsignificant trend toward enhanced student knowledge acquisition. 

## Competing interests

The authors declare that they have no competing interests. 

## Figures and Tables

**Table 1:**
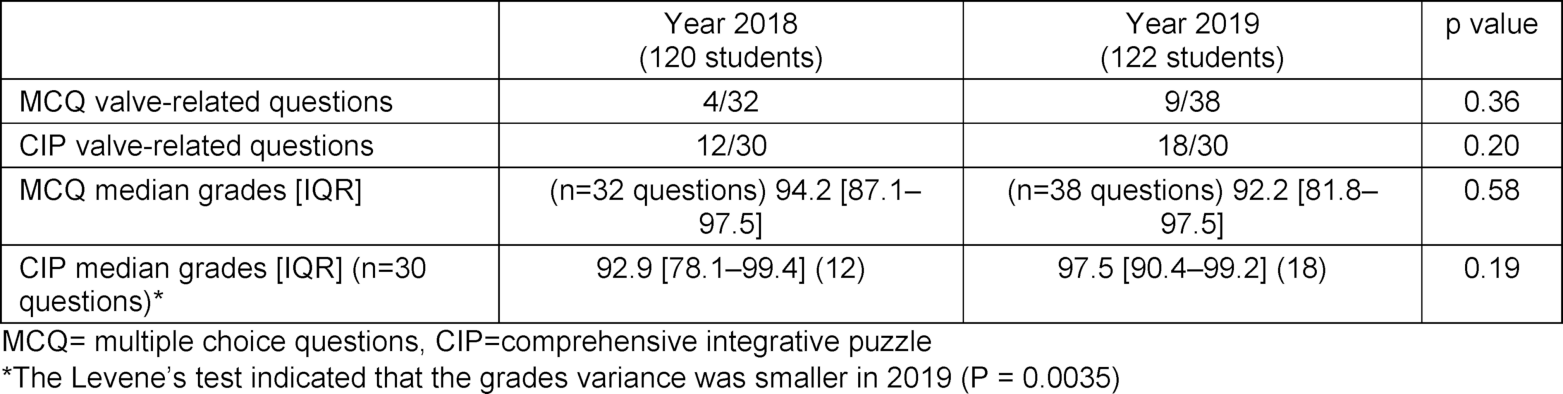
Number of valve-related questions in years 2018-2019 and median grades of tests in all questions

**Table 2:**
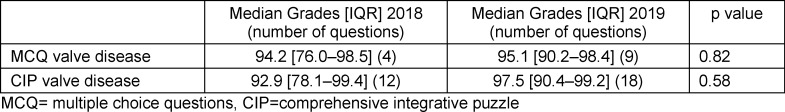
Comparison of grades in valve-related questions between lectures and the two envelopes teaching
